# Understanding HIV Vaccine Misinformation and Vaccine Intentions Among Young Women in South Africa: Insights from an Online Survey

**DOI:** 10.1007/s10461-026-05045-1

**Published:** 2026-03-14

**Authors:** Brendan Maughan-Brown, Teniola I. Egbe, Simamkele Bokolo, Megan Rabin, Candice Chetty-Makkan, Melanie Kornides, Lawrence Long, Sophie Pascoe, Sarah Penuela-Wermers, Sander van der Linden, Harsha Thirumurthy, Alison M. Buttenheim

**Affiliations:** 1 Southern Africa Labour and Development Research Unit (SALDRU), University of Cape Town, Cape Town, South Africa; 2 Department of Medical Ethics and Health Policy, University of Pennsylvania, Philadelphia, USA; 3 Center for Health Incentives and Behavioral Economics, University of Pennsylvania, Philadelphia, USA; 4 Health Economics and Epidemiology Research Office, Faculty of Health Sciences, University of the Witwatersrand, Johannesburg, South Africa; 5 Department of Family and Community Health, University of Pennsylvania, Philadelphia, USA; 6 Department of Global Health, School of Public Health, Boston University, Boston, USA; 7 Department of Psychology, University of Cambridge, Cambridge, UK

**Keywords:** Misinformation, Fake news, HIV vaccine, Vaccines, HIV prevention, Africa

## Abstract

An effective HIV vaccine could significantly reduce HIV incidence, but demand for future HIV vaccines will be affected by misinformation. We conducted an online survey (March-April 2024) to identify the misinformation claims most likely to deter young women in South Africa (*n* = 188) from receiving an HIV vaccine. Participants rated HIV-vaccine misinformation as most concerning (i.e. would make them not want to get vaccinated) and least concerning (i.e. would not change a decision to get vaccinated). From 54 misinformation claims found in peer-reviewed and grey literature (e.g., reports), participants viewed two sets of 9 randomly chosen claims. Within each set, they selected the 3 most concerning and 3 least concerning claims. Claims were ranked according to their likelihood of being selected as most or least concerning. Misinformation claims that were rated as most concerning were about HIV vaccine safety, particularly those suggesting severe adverse health effects (e.g. the vaccine “will kill you”, selected 85% of times viewed). Also rated as concerning were claims that the vaccine was created to harm certain populations (e.g., “designed to sterilise Black women”, 60% selected); that the vaccine increases rape and pregnancies (51% selected); and gives you HIV (50% selected). The least concerning misinformation related to the themes of stigma, vaccine efficacy, and the availability of other HIV prevention options. Misinformation that the HIV vaccine causes harm, increases risky behaviour, and gives recipients HIV may have the greatest impact on vaccination intentions. Research is needed to design and test interventions that build resistance to such misinformation.

**Supplementary Information:**

The online version contains supplementary material available at 10.1007/s10461-026-05045-1.

## Introduction

Nearly 25% of all new HIV infections in sub-Saharan Africa in 2022 occurred among adolescent girls and young women (AGYW) aged 15–24 years [1]. High HIV incidence (8.7 per 1000) among AGYW persists despite widespread access to combination HIV prevention and very high coverage of antiretroviral therapy (ART) among people living with HIV [2]. In South Africa, the country with the largest HIV epidemic [3], HIV prevalence among AGYW is 8.7% [4] – more than two times higher than their male counterparts.

A safe, effective and affordable HIV vaccine remains important for achieving epidemic control [5]. While such a vaccine could enable a profound, rapid, and cost-effective reduction in HIV incidence, its overall success will hinge on high uptake among AGYW and other populations that are vulnerable to HIV. However, as with many vaccines, uptake of future HIV vaccines is likely to be affected by misinformation. In 2019, the World Health Organization recognized vaccine hesitancy as a global health threat, given its impact on vaccine demand [6]. The rollout of COVID-19 vaccines demonstrated that exposure to false or misleading information, often amplified through social media, can substantially reduce vaccine uptake [7–11]. Effective strategies to counter misinformation about HIV vaccines will likely be essential for demand creation when an effective HIV vaccine is eventually discovered and made widely available.

Little is known about the types of misinformation that may influence intentions among AGYW to get vaccinated against HIV. In this paper, we define misinformation as false or misleading information masquerading as legitimate news, regardless of whether it is intended to deceive people [12]. Identifying emerging misinformation claims that are most concerning to AGYW can be useful for developing messages and other communication strategies that can make people more resistant to misinformation when they encounter it in other settings. Such messages can effectively and proactively debunk or “pre-bunk” false and misleading claims about the HIV vaccine and slow the spread of those claims.

This strategy of pre-bunking misinformation is informed by psychological inoculation theory, which posits that informing people of the threat of misinformation, then exposing them to misinformation alongside refutations or explanations that highlight the common fallacies and manipulative tactics used in misinformation, can help build ‘resistance’ to misinformation before it is encountered in the future. Analogous to actual vaccines, if people are exposed to this ‘weakened dose’ of misinformation they will become more immune to misinformation [13, 14]. Inoculation messages can reduce susceptibility to many forms of misinformation [15–17], including countering misinformation about COVID-19 [18–20]. To develop communication strategies that guard against HIV vaccine misinformation, and using methods from previous HPV vaccine research [21], we conducted an online survey toassess which of the misinformation claims that are already circulating about the HIV vaccine aremost concerning to AGYW.

## Methods

### Study Design and Population

We conducted a self-administered survey on Prolific, an online research platform that includes thousands of participants from South Africa. We enrolled participants who met the following criteria: female, aged 18–29 years, resident in South Africa, able to read English, and with an approval rating on Prolific of 95–100% (an indication of high approval of the participant’s submissions by researchers, typically based on successfully completing studies). While using Prolific did not enable us to draw a representative sample (Prolific participants are, for example, more highly educated than the general population of South Africa), it enabled us to rapidly identify concerning claims about the HIV vaccine with a limited budget. For study recruitment, the Prolific platform sent a study invitation to its registered members who were eligible to participate in our study. The message included a link to our survey – which was administered through Qualtrics, an online survey platform. Participants could complete the survey on any device they had access to, and in any location (e.g., at home, school, work, a coffee shop etc.). The study was approved by ethics committees at the University of Pennsylvania, University of the Witwatersrand, Boston University, and University of Cape of Town. Participants provided electronic informed consent.

### Data Collection

We asked participants to rate 54 claims (see Supplementary Table S1) about the HIV vaccine that we considered to be false or misleading (hereafter called ‘misinformation claims’). We identified these claims through a rapid review of published and grey literature written in English. Grey literature is defined as information produced on all levels of government, academia, business and industry in electronic and print formats not controlled by commercial publishing i.e., where publishing is not the primary activity of the producing body [22]. Relevant grey literature included fact sheets, reports, HIV vaccine trials news, or unpublished work, and was identified using the terms: “HIV vaccine concerns”, “HIV vaccine myths” and “HIV vaccine fake information”. Relevant articles with content related to HIV vaccines and published in English in any country were reviewed by two study team members. All misinformation claims relating to an HIV vaccine were extracted. Through an iterative process, duplicate claims were removed and the phrasing of the claims was simplified to be understandable among the study population. Several study team members reviewed and refined the wording of the claims to enhance clarity and accessibility.

In the survey, each participant was exposed to 18 (of a possible 54) randomly selected claims presented to them in two sets consisting of nine claims each. Within the first set of nine randomly selected claims they were asked to select the three claims they found to be ‘most concerning’ about the HIV vaccine. Participants were provided the definition of ‘most concerning’ as ‘it would make you not want to get the HIV vaccine’. From the remaining six claims, we asked participants to select the three claims they found ‘least concerning’ about the HIV vaccine (i.e., ‘it would not change your mind about taking the HIV vaccine’). This process was repeated once more, with participants being shown the second set of nine randomly selected claims from the remaining claims they had not seen in the first round. Each misinformation claim was viewed an average of 63 times (standard deviation: 6.8) with a range of 49–77 for the number of times viewed. Our methods are consistent with previous research assessing the types of Human papillomavirus (HPV) vaccine misinformation that parents find most concerning [21]. At the end of the survey we included a debriefing statement that explained that an HIV vaccine is still under development and not available for use yet, and that the study aimed to understand how false statements and misinformation about the vaccine could change people’s opinions about getting it if it becomes available in the future.

In the survey we also asked an ‘attention check’ question (i.e., what month of the year it was at the time of the survey), and collected data on sociodemographics, HIV status, use of pre-exposure prophylaxis (PrEP) for HIV, COVID-19 vaccination status, and sexual behaviour.

### Statistical Analyses

To determine which claims were most and least concerning, we calculated the number of times each claim was selected, divided by the total number of times it was viewed. For example, if a claim was picked 46 times out of the 55 times it was shown, its selection percentage would be 84% (46/55). Claims with a higher selection percentage were considered more concerning. Claims were ranked according to their likelihood of being selected as most or least concerning. We also stratified the analyses by participants’ self-reported COVID-19 vaccination status, as attitudes and beliefs about vaccination can vary substantially between those who do and do not get vaccinated [10, 11]. To assess patterns in the selection of claims, each claim was categorised by the study team into one of seven themes that were based on the broad topic of each claim (e.g. HIV vaccine safety and HIV vaccine efficacy). All analyses were conducted in STATA 17.0 (StataCorp, College Station, TX).

## Results

Between 18 March 2024 and 11 April 2024, 190 participants completed the survey. We excluded one participant who failed the attention check and one participant who did not complete the online survey. Among 188 participants included in our analyses, the mean age was 25.1 years (Table 1). Participants were predominantly: Black African (89.4%); from Gauteng province (62.7%); self-reported HIV negative (92.0%); currently not taking PrEP (93.6%); had received a COVID-19 vaccine (69.7%); and had had sex in the past 12 months (72.3%). All participants had completed grade 12 schooling (100%) and 84.6% had completed some tertiary education. The percentage who are Black African in our sample aligns roughly with the percentage (85%) of 20–29 South African women who are Black African [23]. However, our sample is more educated than the average South African 25–34 years old, among whom approximately 55% have completed Grade 12 and about 20% have completed some post-school qualification [24].

**Table 1:** Participant characteristics

	Overall (*N* = 188)
Age, mean (SD)	25.1 (2.41)
Race, n (%)	
African/Black	168 (89.4%)
Coloured*	10 (5.3%)
Indian	2 (1.1%)
White	8 (4.3%)
Highest grade you have successfully completed, Grade 12 (Std 10/Matric), n (%)	188 (100%)
Completed any tertiary education, n (%)	
Yes	159 (84.6%)
Province of current residence, n (%)	
Gauteng	118 (62.7%)
KwaZulu-Natal	19 (10.11%)
Western Cape	17 (9.0%)
Eastern Cape	12 (6.4%)
Limpopo	8(4.3%)
Free State	5 (2.7%)
North West	4 (2.1%)
Mpumalanga	4 (2.1%)
Northern Cape	1 (0.5%)
HIV status, negative, n (%)	173 (92.0%)
Currently not on PrEP, n (%)	174 (93.6%)
Got the COVID-19 vaccine, n (%)	131 (69.7%)
Had sex in past 12 months, n (%)	136 (72.3%)
Survey length in minutes, mean (SD)	8.9 (5.47)

*‘Coloured’ is a common and socially acceptable term in South Africa for individuals of mixed race.

### Most and Least Concerning Claims

Table 2 lists the 20 claims most often rated by participants as among the three most or least concerning. Among claims rated as most concerning, those pertaining to the safety of the HIV vaccine, particularly those suggesting severe adverse health effects, were very common. These included claims that the vaccine “will kill you” (selected 85% of times it was shown to participants), that it “will cause liver failure, kidney failure, or heart failure” (82%), and that it “will break down your bone marrow or cause cancer” (78%). Claims relating to effects on children, pregnancy, or fertility were also commonly listed among the three most concerning. Claims that the vaccine was created to harm specific populations also featured in this list (e.g., “designed to sterilise Black women” (60%) and “kill poor people, gay people, sex workers, and Black people” (51%). Thematically, among the 20 claims that were most likely to be rated as one of the three most concerning, 13 (65%) related to vaccine safety, while 3 (15%) related to the vaccine giving a person HIV and 3 (15%) related to harming specific populations.

**Table 2:**
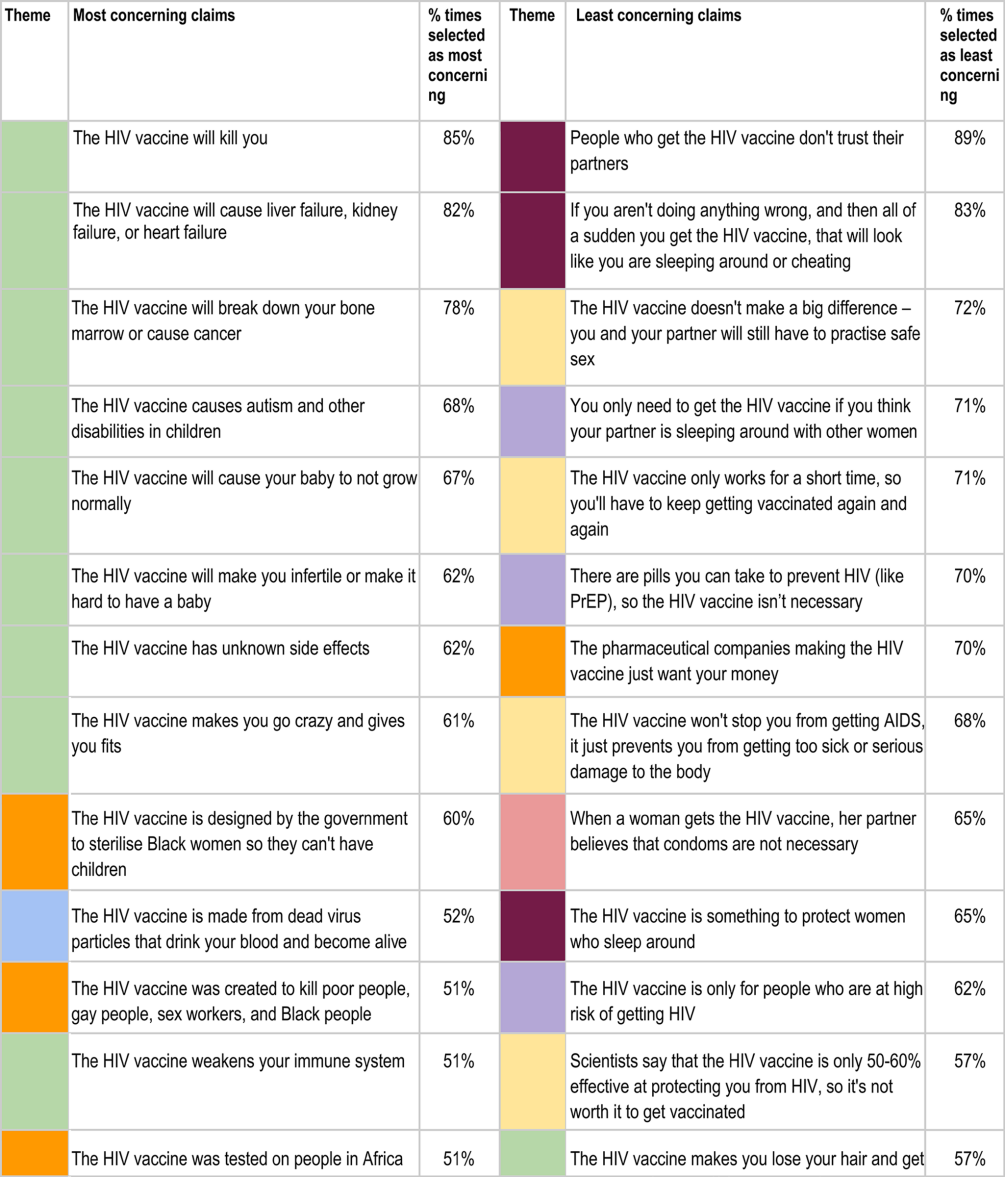

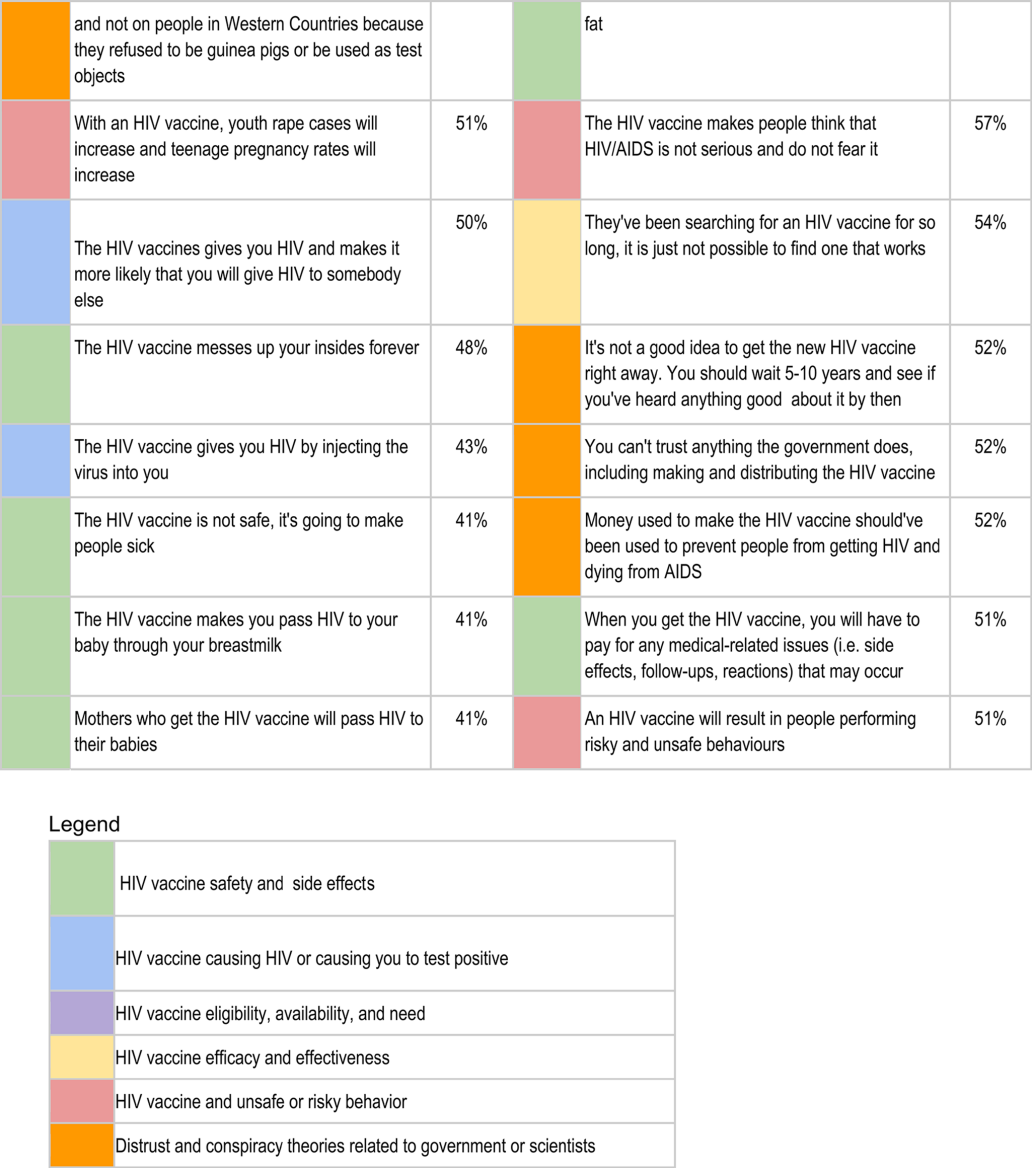
Top 20 most concerning and least concerning claims

Claims that were most likely to be rated as one of the three least concerning were those that pertained to stigma, associations of vaccine use with infidelity, partial vaccine efficacy, and the availability of other HIV prevention options. These included claims that “people who get the HIV vaccine don’t trust their partners” (89%); that “if you aren’t doing anything wrong, and then all of a sudden you get the HIV vaccine, that will look like you are sleeping around or cheating” (83%); and that “the HIV vaccine doesn’t make a big difference – you and your partner will still have to practise safe sex” (72%). The most common theme had to do with partial effectiveness of the vaccine (5 claims, 25%) while the themes of conspiracies and distrust of governments and companies (4 claims, 20%) were the second most common.

Among the sub-groups of 131 participants who had been vaccinated for COVID-19 and 57 participants who were not vaccinated for COVID-19, there were similar patterns in which claims were rated as the most (supplementary Table S2) and least (supplementary Table S3) concerning. In both sub-groups, misinformation claims relating to the safety of the HIV vaccine – primarily extreme adverse events - predominated as the most concerning.

## Discussion

This study used an online survey to examine young South African women’s reactions to existing false claims about HIV vaccines, identifying and categorizing those they found most and least concerning. The claims we presented to participants were drawn from a review of published and grey literature on efforts to develop and test HIV vaccines. We found that misinformation relating to the safety of the vaccine was most concerning for participants, especially claims suggesting that the vaccine has severe adverse events leading to death and those relating to childbearing. In contrast, claims that related to potential HIV vaccines having only partial efficacy, or their use being associated with stigma and infidelity, were less concerning to participants.

Early identification of impactful misinformation is important for several reasons. Misinformation will almost certainly reduce demand for any HIV vaccine that becomes available in the future, as has been the case for other vaccines such as those for COVID-19 [7–11], and Human papillomavirus (HPV) [25]. Moreover, even when misinformation is prevalent, some claims may be more impactful than others [26]. Identifying the specific misinformation claims that intended users of HIV vaccines find most concerning is essential for developing targeted interventions to protect against those claims, particularly since it is not feasible to address all misinformation claims that may circulate in communities and on social media. These claims may be especially well suited for “pre-bunking” interventions or campaigns – i.e. the practice of exposing people to misinformation before they encounter it, while also explaining why the claims are false. In other contexts, this approach has been shown to reduce the credibility of misinformation [27–29], dampen the effect of misinformation on intentions and behaviours [30], and reduce its spread [19, 27]. Pre-bunking is an important strategy because of the continued influence effect: people often continue to be influenced by misinformation once exposed to it, even after attempts have been made to debunk or correct the misinformation [14, 31, 32].

In our study, misinformation relating to the safety of the vaccine was the most concerning for participants. These findings align with research showing that vaccine safety concerns reduced the uptake of a COVID-19 vaccine [10], and are among the top reasons for HPV vaccination refusal [33]. In South Africa, a study found that nearly 40% of those most hesitant to receive the COVID-19 vaccine believed it could be fatal [11], illustrating the high prevalence of erroneous beliefs about adverse health consequences of vaccination. In contexts such as COVID-19 where many people were vaccinated over a short period of time, these beliefs may emerge given the likelihood that some deaths, unrelated to COVID-19, inevitably occur soon after vaccination and become mistakenly associated with it. This underscores the importance of interventions to dispel this type of misinformation. Moreover, individuals who deliberately spread misinformation about vaccines often focus on claims about severe health consequences [34]. Aligned with our findings, the myth that a vaccine causes infertility is also common to many other vaccines [35–38].

Misinformation claims based on distrust, and conspiracy theories related to government and scientists, were also a significant concern to participants. Often, the claims related to discrimination and the targeting of specific groups: people in Africa, Black women, homosexuals, sex workers and poor people. The use of conspiracy theories is a common tactic to create and spread misinformation [39], and has been demonstrated to increase vaccine hesitancy and refusal [40]. Pre-bunking misinformation based on conspiracy theories has been a central component of many successful pre-bunking interventions such as Bad News, Cat Park and Go Viral! [17, 19, 27]. Our findings align with prior studies on misinformation and suggest that the early detection and pre-bunking of conspiracy-based misinformation may be important for a future HIV vaccine rollout.

The third type of misinformation that young women found concerning was the claim that an HIV vaccine would give people HIV. The belief that a vaccine causes the condition it is designed to prevent is common to many vaccines, including COVID-19 [41, 42], and influenza [43]. Two aspects of vaccines make this a commonly held myth that should be anticipated in future vaccination efforts. First, many vaccines contain either live or inactive bacteria or viruses and this leads to confusion. Second, some side-effects of vaccines are similar to the symptoms of the disease being vaccinated against, which can reinforce the myth that the vaccine causes the disease.

While demand for a future HIV vaccine will likely be influenced by perceptions of vaccine safety and efficacy (e.g. previous studies show that beliefs of vaccine effectiveness predicted COVID-19 vaccine uptake [10]), participants in our study were less concerned about claims that had to with an HIV vaccine being only partially effective. Ultimately, the decision to vaccinate will be influenced by weighing the costs and benefits. If the perceived safety risks associated with a vaccine are very high (i.e., death or serious harm) then it is unlikely that the perceived benefits of getting vaccinated will motivate vaccination. On the other hand, a study showed that among a sample of people who were concerned about the safety of COVID-19 vaccines, it was perceived efficacy that predicted vaccine uptake [10] – thus indicating that if the perceived benefits are high enough then people may be willing to accept some perceived safety risk. Misinformation relating to stigma and whether an HIV vaccine is needed were also among the least concerning claims. Reviews of misinformation associated with different vaccines often find claims that the vaccine is not necessary (e.g., because of natural remedies, or because of natural immunity), but vaccine misinformation claims related to stigma are not often found to be a major theme [36, 44]. It is possible that when an HIV vaccine is available the potential for stigma may become more salient given the high levels of perceived HIV-related stigma among young people in South Africa [45], and related misinformation could be of greater concern.

Compared to people less hesitant about vaccination in general, those very hesitant often have quite different beliefs about the risks and benefits of vaccines [10, 11]. We therefore anticipated that study participants who had been vaccinated for COVID-19 would be concerned by different misinformation claims than those not vaccinated for COVID-19. Overall, we did not find this to be the case. Findings suggest that some types of vaccine misinformation – in particular relating to safety concerns – may be impactful regardless of vaccination history. This suggests that the rapid targeting of specific misinformation could be impactful across the population, regardless of prior choices.

This study has several limitations. First, participants were more highly educated than the average South African population of 18–29 year-old women. The extent to which study results generalise to other young women, and more widely, is unknown. Other studies have shown that beliefs about vaccines, and reasons for not getting vaccinated, can vary based on the believers’ socioeconomic and demographic characteristics [11]. This suggests there is a need for research that informs population-specific tailoring of campaigns to mitigate the impact of misinformation about the HIV vaccine. Second, our survey did not ask explicitly about future vaccine intentions since an HIV vaccine is not currently available. Instead, we defined claims that are concerning as those that may make people not want to get the HIV vaccine. Some participants may find certain claims concerning, but this might not ultimately affect their intentions to get vaccinated. Third, our study presented a hypothetical future HIV vaccine scenario. The degree to which claims are found to be of concern to participants could shift as the decisions and behaviours relating to an HIV vaccine become more relevant and change in salience.

In conclusion, the greatest impact on vaccination intentions in South Africa may come from misinformation that the HIV vaccine results in severe adverse health consequences, was designed to harm certain people, increases harmful behaviours, and gives people HIV. Research is needed to design and test interventions to build resistance to such misinformation and ensure high demand for the HIV vaccine. Our results also underscore the value of research that identifies the most impactful misinformation claims that emerge during the early stages of a vaccine rollout and develops targeted interventions to counter them.

## Supplementary Information

Below is the link to the electronic supplementary material.

Supplementary Material 1

## Data Availability

Data are available on request to the corresponding author.
