# Supplementary material for: Understanding HIV Vaccine Misinformation and Vaccine Intentions Among Young Women in South Africa: Insights from an Online Survey
Source: AIDS Behav. 2026 Mar 14;30(7):2202–10. doi: 10.1007/s10461-026-05045-1 (PMC13400596; doi:10.1007/s10461-026-05045-1)
Supplement: Supplementary file 1 — Supplementary Material 1 [file 10461_2026_5045_MOESM1_ESM.docx]

**Supplementary File 1**

**Article:** Understanding HIV vaccine misinformation and vaccine intentions among young women in South Africa: insights from an online survey

Table S1: Full list of claims in alphabetical order

| An HIV vaccine will result in people performing risky and unsafe behaviours |
| --- |
| Getting the HIV vaccine makes you test positive on HIV tests |
| Getting the HIV vaccine means you are HIV-positive |
| If you aren't doing anything wrong, and then all of a sudden you get the HIV vaccine, that will look like you are sleeping around or cheating |
| It's not a good idea to get the new HIV vaccine right away. You should wait 5-10 years and see if you've heard anything good about it by then |
| Money used to make the HIV vaccine should've been used to prevent people from getting HIV and dying from AIDS |
| Mothers who get the HIV vaccine will pass HIV to their babies |
| People who get an HIV vaccine think they don't have to use condoms and they can sleep with whoever they want |
| People who get the HIV vaccine don't trust their partners |
| Scientists say that the HIV vaccine is only 50-60% effective at protecting you from HIV, so it's not worth it to get vaccinated |
| The HIV vaccine causes autism and other disabilities in children |
| The HIV vaccine doesn't make a big difference – you and your partner will still have to practice safe sex |
| The HIV vaccine gives you HIV by injecting the virus into you |
| The HIV vaccine has existed for a long time but was hidden from people who need it so they can die of AIDS |
| The HIV vaccine has lots of side effects: you could get a rash, sleeplessness, fever, loss of appetite, be extremely tired, and all kinds of things |
| The HIV vaccine has unknown side effects |
| The HIV vaccine is designed by the government to sterilise Black women so they can't have children |
| The HIV vaccine is just like the COVID-19 vaccine, it will change your DNA permanently |
| The HIV vaccine is made from dead virus particles that drink your blood and become alive |
| The HIV vaccine is not safe, it's going to make people sick |
| The HIV vaccine is only for people who are at high risk of getting HIV |
| The HIV vaccine is so expensive that people without money can't get it |
| The HIV vaccine is something to protect women who sleep around |
| The HIV vaccine makes people think that HIV/AIDS is not serious and do not fear it |
| The HIV vaccine makes you go crazy and gives you fits |
| The HIV vaccine makes you lose your hair and get fat |
| The HIV vaccine makes you pass HIV to your baby through your breastmilk |
| The HIV vaccine messes up your insides forever |
| The HIV vaccine only works for a short time, so you'll have to keep getting vaccinated again and again |
| The HIV vaccine was created to kill poor people, gay people, sex workers, and black people |
| The HIV vaccine was tested on people in Africa and not on people in Western Countries because they refused to be guinea pigs or be used as test objects |
| The HIV vaccine weakens your immune system |
| The HIV vaccine will break down your bone marrow or cause cancer |
| The HIV vaccine will cause liver failure, kidney failure, or heart failure |
| The HIV vaccine will cause your baby to not grow normally |
| The HIV vaccine will kill you |
| The HIV vaccine will make you infertile or make it hard to have a baby |
| The HIV vaccine won't stop you from getting AIDS, it just prevents you from getting too sick or serious damage to the body |
| The HIV vaccines give you HIV and makes it more likely that you will give HIV to somebody else |
| The pharmaceutical companies making the HIV vaccine just want your money |
| The vaccine will mess up your body - you will grow another finger or something |
| There are pills you can take to prevent HIV (like PrEP), so the HIV vaccine is not necessary |
| There have already been vaccines like the HIV vaccine that made people die |
| There is actually an HIV vaccine already but the pharmaceutical companies are hiding it so they can keep making money on HIV treatment |
| There is treatment for HIV, so the HIV vaccine is not necessary |
| There will be a new variant of HIV virus in the next 5 years, so the vaccine you get won't protect against that variant |
| They've been searching for an HIV vaccine for so long, it is just not possible to find one that works |
| When a woman gets the HIV vaccine, her partner believes that condoms are not necessary |
| When you get the HIV vaccine, you will have to pay for any medical-related issues (i.e. side effects, follow-ups, reactions) that may occur |
| With an HIV vaccine, youth rape cases will increase and teenage pregnancy rates will increase |
| You can't trust anything the government does, including making and distributing the HIV vaccine |
| You can't trust what scientists and doctors tell you is in the HIV vaccine |
| You only need to get the HIV vaccine if you think your partner is sleeping around with other women |
| You should be careful of things like the HIV vaccine that don't really cure HIV. Maybe it just helps you live longer with the symptoms so you keep spending money on medicines |

Table S2: Top 20 most concerning claims by COVID-19 vaccination status

| **Vaccinated** for COVID-19 | | | **Unvaccinated** for COVID-19 | | |
| --- | --- | --- | --- | --- | --- |
| Theme | Most concerning | **% times selected** | Theme | Most concerning | **% times selected** |
|  | The HIV vaccine will cause liver failure, kidney failure, or heart failure | 89% |  | The HIV vaccine will kill you | 80% |
|  | The HIV vaccine will kill you | 87% |  | The HIV vaccine weakens your immune system | 79% |
|  | The HIV vaccine will break down your bone marrow or cause cancer | 85% |  | The HIV vaccine will cause your baby to not grow normally | 76% |
|  | The HIV vaccine will make you infertile or make it hard to have a baby | 71% |  | The HIV vaccine causes autism and other disabilities in children | 74% |
|  | The HIV vaccine causes autism and other disabilities in children | 67% |  | The HIV vaccine makes you go crazy and gives you fits | 67% |
|  | The HIV vaccine is designed by the government to sterilise Black women so they can't have children | 66% |  | The HIV vaccine has unknown side effects | 67% |
|  | The HIV vaccine will cause your baby to not grow normally | 63% |  | The HIV vaccine will break down your bone marrow or cause cancer | 65% |
|  | The HIV vaccine has unknown side effects | 60% |  | The HIV vaccine is made from dead virus particles that drink your blood and become alive | 61% |
|  | With an HIV vaccine, youth rape cases will increase and teenage pregnancy rates will increase | 58% |  | The HIV vaccine will cause liver failure, kidney failure, or heart failure | 57% |
|  | The HIV vaccine makes you go crazy and gives you fits | 56% |  | The HIV vaccine was created to kill poor people, gay people, sex workers, and black people | 53% |
|  | The HIV vaccine was created to kill poor people, gay people, sex workers, and black people | 51% |  | The HIV vaccine is not safe, it's going to make people sick | 50% |
|  | The HIV vaccine was tested on people in Africa and not on people in Western Countries because they refused to be guinea pigs or be used as test objects | 51% |  | The HIV vaccine is designed by the government to sterilise Black women so they can't have children | 47% |
|  | The HIV vaccines give you HIV and makes it more likely that you will give HIV to somebody else | 50% |  | The HIV vaccines give you HIV and makes it more likely that you will give HIV to somebody else | 47% |
|  | The HIV vaccine messes up your insides forever | 50% |  | The vaccine will mess up your body - you will grow another finger or something | 47% |
|  | The HIV vaccine gives you HIV by injecting the virus into you | 48% |  | The HIV vaccine was tested on people in Africa and not on people in Western Countries because they refused to be guinea pigs or be used as test objects | 47% |
|  | The HIV vaccine is made from dead virus particles that drink your blood and become alive | 48% |  | The HIV vaccine will make you infertile or make it hard to have a baby | 45% |
|  | Mothers who get the HIV vaccine will pass HIV to their babies | 47% |  | There will be a new variant of HIV virus in the next 5 years, so the vaccine you get won't protect against that variant | 43% |
|  | There have already been vaccines like the HIV vaccine that made people die | 44% |  | The HIV vaccine messes up your insides forever | 42% |
|  | The HIV vaccine is so expensive that people without money can't get it | 44% |  | Scientists say that the HIV vaccine is only 50-60% effective at protecting you from HIV, so it's not worth it to get vaccinated | 40% |
|  | There is actually an HIV vaccine already but the pharmaceutical companies are hiding it so they can keep making money on HIV treatment | 42% |  | The HIV vaccine makes you pass HIV to your baby through your breastmilk | 39% |

Legend

|  | HIV vaccine safety and side effects |
| --- | --- |
|  | HIV vaccine causing HIV or causing you to test positive |
|  | HIV vaccine eligibility, availability, and need |
|  | HIV vaccine efficacy and effectiveness |
|  | HIV vaccine and unsafe or risky behaviour |
|  | Distrust and conspiracy theories related to government or scientists |
|  | HIV and HIV-vaccine related stigma |

Table S3: Top 20 least concerning claims by COVID-19 vaccination status

| **Vaccinated** for COVID-19 | | | **Unvaccinated** for COVID-19 | | |
| --- | --- | --- | --- | --- | --- |
| Theme | Least concerning | **% times selected** | Theme | Least concerning | **% times selected** |
|  | People who get the HIV vaccine don't trust their partners | 95% |  | The HIV vaccine only works for a short time, so you'll have to keep getting vaccinated again and again | 91% |
|  | If you aren't doing anything wrong, and then all of a sudden you get the HIV vaccine, that will look like you are sleeping around or cheating | 87% |  | If you aren't doing anything wrong, and then all of a sudden you get the HIV vaccine, that will look like you are sleeping around or cheating | 77% |
|  | The HIV vaccine doesn't make a big difference – you and your partner will still have to practise safe sex | 76% |  | People who get the HIV vaccine don't trust their partners | 76% |
|  | You only need to get the HIV vaccine if you think your partner is sleeping around with other women | 73% |  | People who get an HIV vaccine think they don't have to use condoms and they can sleep with whoever they want | 73% |
|  | Scientists say that the HIV vaccine is only 50-60% effective at protecting you from HIV, so it's not worth it to get vaccinated | 70% |  | There are pills you can take to prevent HIV (like PrEP), so the HIV vaccine is not necessary | 73% |
|  | The pharmaceutical companies making the HIV vaccine just want your money | 69% |  | The pharmaceutical companies making the HIV vaccine just want your money | 68% |
|  | The HIV vaccine won't stop you from getting AIDS, it just prevents you from getting too sick or serious damage to the body | 68% |  | You only need to get the HIV vaccine if you think your partner is sleeping around with other women | 67% |
|  | There are pills you can take to prevent HIV (like PrEP), so the HIV vaccine is not necessary | 68% |  | The HIV vaccine won't stop you from getting AIDS, it just prevents you from getting too sick or serious damage to the body | 67% |
|  | When a woman gets the HIV vaccine, her partner believes that condoms are not necessary | 66% |  | The HIV vaccine makes you lose your hair and get fat | 64% |
|  | The HIV vaccine is something to protect women who sleep around | 65% |  | There will be a new variant of HIV virus in the next 5 years, so the vaccine you get won't protect against that variant | 63% |
|  | The HIV vaccine only works for a short time, so you'll have to keep getting vaccinated again and again | 65% |  | When you get the HIV vaccine, you will have to pay for any medical-related issues (i.e. side effects, follow-ups, reactions) that may occur | 63% |
|  | The HIV vaccine is only for people who are at high risk of getting HIV | 64% |  | The HIV vaccine is only for people who are at high risk of getting HIV | 62% |
|  | The vaccine will mess up your body - you will grow another finger or something | 59% |  | When a woman gets the HIV vaccine, her partner believes that condoms are not necessary | 62% |
|  | It's not a good idea to get the new HIV vaccine right away. You should wait 5-10 years and see if you've heard anything good about it by then | 57% |  | The HIV vaccine doesn't make a big difference – you and your partner will still have to practice safe sex | 62% |
|  | The HIV vaccine makes people think that HIV/AIDS is not serious and do not fear it | 57% |  | The HIV vaccine has existed for a long time but was hidden from people who need it so they can die of AIDS | 62% |
|  | They've been searching for an HIV vaccine for so long, it is just not possible to find one that works | 55% |  | The HIV vaccine is something to protect women who sleep around | 61% |
|  | The HIV vaccine makes you lose your hair and get fat | 55% |  | An HIV vaccine will result in people performing risky and unsafe behaviours | 60% |
|  | The HIV vaccine is so expensive that people without money can't get it | 55% |  | You can't trust anything the government does, including making and distributing the HIV vaccine | 59% |
|  | You should be careful of things like the HIV vaccine that don't really cure HIV. Maybe it just helps you live longer with the symptoms so you keep spending money on medicines | 51% |  | There is actually an HIV vaccine already but the pharmaceutical companies are hiding it so they can keep making money on HIV treatment | 57% |
|  | You can't trust anything the government does, including making and distributing the HIV vaccine | 51% |  | Money used to make the HIV vaccine should've been used to prevent people from getting HIV and dying from AIDS | 56% |

Legend

|  | HIV vaccine safety and side effects |
| --- | --- |
|  | HIV vaccine causing HIV or causing you to test positive |
|  | HIV vaccine eligibility, availability, and need |
|  | HIV vaccine efficacy and effectiveness |
|  | HIV vaccine and unsafe or risky behaviour |
|  | Distrust and conspiracy theories related to government or scientists |
|  | HIV and HIV-vaccine related stigma |
